# Identification of viral etiology of acute respiratory tract infections in children and adults in Tabanan, Bali, Indonesia

**DOI:** 10.1099/acmi.0.000120

**Published:** 2020-03-25

**Authors:** Ni Wayan Widhidewi, Ageng Wiyatno, Aghnianditya Kresno Dewantari, Lila Paramasatiari, Sri Agung Aryastuti, I Nengah Artika, Wayan Doddy Setiawan, Amin Soebandrio, Khin Saw Aye Myint, Dodi safari

**Affiliations:** ^1^​ Faculty of Medicine, Universitas Indonesia, Jl. Salemba Raya, No. 6 Jakarta, Indonesia; ^2^​ Faculty of Medicine, Universitas Warmadewa, Jl. Terompong No.24 Denpasar, Bali, Indonesia; ^3^​ Eijkman Institute of Molecular Biology, Jl. Diponegoro No. 69, Jakarta, Indonesia; ^4^​ Tabanan General Hospital, Jl. Pahlawan, Tabanan, Bali, Indonesia

**Keywords:** Acute respiratory tract infection, etiology, virus, Bali

## Abstract

Acute respiratory tract infection (ARTI) is the most common infectious disease in humans worldwide. The morbidity and mortality rates are high, especially in developing countries from Southeast Asia and Africa. While ARTI is commonly associated with viruses, there is limited data on the spectrum of viruses causing ARTI in developing countries, including Indonesia. This study was based on utilizing molecular techniques targeting a panel of 11 endemic and emerging respiratory viral pathogens including zoonotic viruses in a cohort of children and adults presenting at Tabanan General Hospital, Bali, with acute respiratory illness, from January to November 2017. In total, 98 out of 200 samples (49.0 %) tested positive for viruses. Our study confirmed 64.3 % viral etiology in children and 12.2 % in adults. Viruses that were detected were *Herpesviridae* (15.0 %) followed by enteroviruses (12.0 %), influenza A virus (11.5 %), respiratory syncytial virus (8.0 %), *Adenoviridae* (6.5 %), human metapneumovirus (3.5 %), *Paramyxoviridae* (2.0 %), bocavirus (1.0 %) and *Coronaviridae* (0.5 %). The study sheds light on the viral spectrum of ARTI in children and adults in Tabanan, Bali, Indonesia

Acute respiratory tract infection (ARTI), the most common infectious disease in humans, is a growing global health problem [1, 2]. It is easily transmitted between humans, and known to cause high morbidity and mortality in all age groups [3]. ARTI is predicted to be the cause of 4.5 million deaths every year worldwide [4]. In 2015, pneumonia was responsible for 15 % of all deaths of children under 5 years old [4, 5] with most of the cases occurring in developing countries especially in Africa and Southeast Asia [2, 5, 6].

In Indonesia, ARTI is reported as one of the top five common diseases associated with high mortality in children and adults. According to the Republic of Indonesia Health System Review in 2013 [7], ARTI is ranked second after prematurity in causing death in children under 5 years. Whereas in adults, lower respiratory tract infection is the fourth cause of death after stroke, ischemic heart disease and diabetes mellitus. Altogether respiratory infections accounted for the death of 81 100 Indonesians in 2012 [4]. Respiratory viruses including influenza A and B, respiratory syncytial virus (RSV), adenovirus, and parainfluenza virus are the most frequently detected viruses in patients with ARTI in Southeast Asia [8]. On the other hand, new emerging viruses are on the rise, such as human metapneumovirus (HMPV) and human bocavirus [3, 9]. In this study, throat swab specimens were collected from patients with respiratory symptoms to identify viral etiological agents of ARTI. The data will provide insights on the epidemiology of respiratory viruses in subjects with ARTI in Tabanan region, Bali, Indonesia.

Throat swab specimens were collected from 200 out-patients admitted to Tabanan General Hospital, a regency hospital in Tabanan regency, Bali, between January to November 2017. Patients with symptoms of ARTI with an onset of illness ≤7 days were included in the study. The enrollment criteria included fever (≥37.5 °C) or history of fever with one or more of the following respiratory symptoms: cough, rhinorrhea, nasal obstruction and sore throat. Data on demography, risk factors, exposure to wild animals and clinical symptoms were also collected on day of admission by trained hospital staff.

Throat swabs from patients were preserved in 2 ml of Viral Transport Medium (VTM) prepared in-house, containing bovine brain heart infusion and antibiotics. Specimens were immediately stored at −20 °C at the hospital. Every two days, the specimens were transported to the Biology Molecular Laboratory, Faculty of Medicine, University of Warmadewa, Bali on ice and stored at −80 °C. Samples in batches of 50 were transported using dry ice to the Eijkman Institute for Molecular Biology laboratory in Jakarta and stored at −80 °C prior to testing.

Viral nucleic acid was extracted using QIAamp Viral RNA Minikit (Qiagen, Hilden, Germany) according to the manufacturer's instructions. Viral specific targets were identified by using reverse transcription–PCR (RT-PCR). In summary, 60 µl of viral DNA-RNA was obtained and 4 µl used as a template for complementary DNA (cDNA) synthesis using GoScript Reverse Transcription System (Promega, Madison, USA) and random hexamers.

Singleplex PCR assays were used for detection of a panel of respiratory viruses using family-level primers for *Paramyxoviridae*, *Herpesviridae, Coronaviridae*, *Hantaviridae*, *Adenoviridae, Arenaviridae;* genus-level primers for *Enterovirus, Henipavirus*, *Influenza A virus*, *Bocavirus*; and *Pneumovirinae* sub-family primer including respiratory syncytial virus (RSV) and human metapneumovirus (HMPV). All of the primers and positive controls that were used in the amplification reaction were based on previous reports [10–17]. Singleplex PCR reaction was performed in thermal cycler ProFlex PCR System with appropriate run controls. A recombinant plasmid representing sequence fragments of all family viruses was constructed and used as the positive control. For amplification, 2 µl of cDNA template was added to 23 µl of Promega Go Taq Green Polymerase Master Mix (Promega, Madison, USA). All PCR products were analysed using electrophoresis in 1.5 % agarose gel. Visualization of positive band was performed using Gel Imaging BioRad Gel Doc XR System and Quantity One 1-D Analysis Software (Bio-Rad, California, USA). All samples with a positive band were followed up for further characterization by fragment sequencing based on Sanger method using BigDye Terminator v3.1 and Applied Biosystem (ABI) sequencing machine. Sequencing results were analysed using Geneious Software R8 version 8.1 (Biomatters Ltd, Auckland, New Zealand) and compared with GenBank database by blast for sequence homology.

In this study, the age of patients ranged from 8 months to 80 years, with a median 9 years. Among the patients enrolled, 112 (56.0 %) were males. Children (age group 0–17 years) accounting for 159 (79.5 %) and adults (age group ≥18 years) for 41 (20.5 %) (Table 1). Most patients had fever less than five days (89.5 %) with 191 (95.5 %) of patients developing fever before admission and 79 (39.5 %) at admission. The respiratory symptoms at admission were tabulated in Table 1. Among 200 throat swabs tested, 98 samples (49.0 %) tested positive for viruses (Table 2). We found that viral detection rate in children (64.3 %) was significantly higher than adults (35.7 %), (*P*=0.005). Meanwhile, 57.1 % of virus positive patients were males and there was no association with onset of fever, travel history, and contact with animals. Majority of virus positive patients had symptoms of cough (89.8 %) and runny nose (67.3 %). In this study, viral detection rate was highest with *Herpesviridae* (15.0 %), followed by *Enteroviridae* (12.0 %), *Influenza A virus* (11.5 %), RSV (8.0 %), *Adenoviridae* (6.5 %), HMPV (3.5 %), *Paramyxoviridae* (2.0 %), *Bocaviridae* (1.0 %) and *Coronaviridae* (0.5 %) (Table 2).

**Table 1.** Patient information and clinical presentation of acute respiratory tract infections enrolled in Tabanan General Hospital, Bali

Characteristic	Number of cases (%) (*n*=200)	Number (%) of positive for viruses (*n*=98)
Sex, male	112 (56.0)	56 (57.1)
Age (year)		
<6	94 (47.0)	63 (67.7)
6–17	65 (32.5)	23 (24.2)
>18	41 (20.5)	12 (28.6)
Onset of fever (<5 days)	179 (89.5)	85 (86.7)
Fever before admission	191 (95.5)	92 (93.9)
Fever on admission	79 (39.5)	34 (34.7)
Travel history	5 (2.5)	3 (3.1)
Contact with animals	22 (11.0)	7 (7.1)
Chills	78 (39.0)	30 (30.6)
Headache	102 (51.0)	35 (35.7)
Dizziness	114 (57.0)	48 (49.0)
Vomiting	88 (44.0)	44 (44.9)
Nausea	97 (48.5)	39 (39.8)
Sore throat	89 (44.5)	36 (36.7)
Cough	183 (91.5)	88 (89.8)
Runny nose	123 (61.5)	66 (67.3)
Difficulty in breathing	10 (5.0)	5 (5.1)
Rash	18 (9.0)	6 (6.1)
Malaise	39 (19.5)	16 (16.3)
Lost of appetite	28 (14)	14 (14.3)

**Table 2.** Viral detection from 200 patients with suspected acute respiratory tract infection enrolled in Tabanan General Hospital, Bali

Viruses detected	n	%
Single virus (specimen)	81	40.5
Co-infection viruses (specimen)	17	8.5
*Dual infection*		
Influenza; CMV	3	1.5
HMPV; CMV	3	1.5
RSV; CMV	2	1
Adenovirus; CMV	2	1
RSV-B; Enterovirus 84	1	0.5
Bocavirus; CMV	1	0.5
Rhinovirus C; Influenza	1	0.5
Influenza H1N1; CMV	1	0.5
*Triple infection*		
Coxsackievirus A24; Influenza; RSV	1	0.5
Rhinovirus C; Adenovirus C; CMV	1	0.5
*Quadruple infection*		
Rhinovirus C; CMV; RSV; Influenza	1	0.5
*Coronaviridae*	1	0.5
Human Coronavirus OC43	1	0.5
Influenza A virus	23	11.5
H3N2	14	7
H1N1	8	4
H1N2	1	0.5
Enterovirus	24	12
Enterovirus 84	1	0.5
Coxsackievirus A6	1	0.5
Coxsackievirus A24	1	0.5
Rhinovirus A	10	5
Rhinovirus C	10	5
Coxsackievirus B3	1	0.5
*Herpesviridae*	30	15
CMV	28	14
Adenovirus	13	6.5
Human Adenovirus-B2	4	2
Human Adenovirus-B3	8	4
Human Adenovirus-C	1	0.5
*Paramyxovirinae*	4	2
Measles D8	1	0.5
Parainfluenza virus 3	3	1.5
Bocavirus	2	1
Respiratory syncytial virus	16	8
RSV A	12	6
RSV B	4	2
Human metapneumovirus	7	3.5

CMV, Cytomegalovirus; HMPV, Human Metapneumo Virus; RSV, Respiratory Syncytial Virus.

Almost all of the *Herpesviridae* were further characterized to be cytomegalovirus (CMV) (*n*=28, 14.0 %). In this study, CMV was the most prevalent among children and *Enteroviridae* was the most prevalent among adults (Table 2). The subtypes of the influenza A virus were identified as H3N2 (*n*=14, 7.0 %), H1N1 (*n*=8, 4.0 %), and H1N2 (*n*=1, 0.5 %) (Table 2). Majority of enteroviruses were characterized as rhinovirus A (*n*=10, 5.0 %) and rhinovirus C (*n*=10, 5.0 %) followed by enterovirus 84, coxsackievirus A6, coxsackievirus A24, and coxsackievirus B3 with each of them accounting for 0.5 % (n=1). Both strains of RSV were detected in this study, RSV A (*n*=12, 6.0 %) and RSV B (*n*=4, 2.0 %). The *Adenoviridae* positives were found to be human adenovirus B2 (n=4, 2.0 %), B3 (n=8, 4.0 %) and C (n=1, 0.5 %).

Single virus detection was observed in 40.5 % samples (*n*=81), and co-detection in 7.0 % (*n*=14) for two viruses, 1.0 % (*n*=2) for three viruses and 0.5 % (*n*=1) for four viruses. Co-detection of influenza-CMV (*n*=3) and HMPV-CMV (*n*=3) were the most common co-detection identified in this study. Among 17 cases of co-detection in this study, 85.7 % were from children with the majority of under 6 years age group.

The study identified the epidemiology of respiratory virus infections in patients presenting to a regency hospital in Bali, Indonesia. Bali is one of the most popular domestic and international tourist destinations in Indonesia which has a significant role for the country’s economics and healthcare. The virus detection rate of 49.0 % from the total number of samples was similar to those previously reported [18–21]. Although the specimens were collected during the acute phase of the disease following strict eligibility criteria, more than 50.0 % of patients remain undiagnosed which was likely to be associated with bacteria or other viruses not included in our panels [22]. As reported in an earlier study, the virus detection rate was significantly higher in children than adults as they are more prone to ARTI because of their immature immune system and lack of hygiene [22, 23]. In our study, 16 (8.0 %) patients were RSV-positive and more frequently in children under 5 years old (*n=*15, 7.5 %). Although RSV outbreaks are known to vary from year to year according to geographical pattern, majority of RSV infection in this study was observed in March during the height of the rainy season which is consistent with earlier studies in tropical countries [24, 25].

Using RSV PCR panel, we also detected seven HMPV all in children with three of them developing bronchopneumonia (data not shown). HMPV is known to pose significant threat causing upper and lower respiratory illness in children and elderly, especially those with underlying respiratory and immunocompromised conditions [26, 27]. Two genotypes of HMPV have been identified to date, which can be classified into five lineages, A1, A2a, A2b, B1 and B2. However, their association with disease severity remains unclear [28, 29]. There was only a single report on HMPV from Indonesia documenting the association of HMPV lineage A1 and A2 with asthma exacerbations and pneumonia [30] indicating that HMPV should be screened in those with chronic and severe respiratory infection. Such data on clinically important pathogens could become critical to implement vaccines once they become available.

Further characterization of positive enterovirus specimens revealed one coxsackievirus A24 subtype (0.5 %) that is known to be associated with acute hemorrhagic conjunctivitis outbreaks [31, 32] and acute flaccid paralysis [33]. Coxsackievirus A6 was commonly linked with respiratory infection, herpangina and hand, foot and mouth disease (HFMD) outbreaks [34, 35]. In one study, the virus was observed in a patient with lower respiratory tract infection [36]. CMV, although not a respiratory pathogen, was detected in a number of cases (14 %) in our study, and is most likely associated with a suppressed immune system. In addition, the majority of the coinfection were associated with CMV, which might be a reinactivation rather than an etiological agent.

Studies have reported co-detection rates of respiratory viruses ranging from 7 to 40 % [18, 35, 37]. Co-detection was observed in the form of influenza-CMV and HMPV-CMV in 8.5 % of specimens especially in children under the age of 6 years, none of which were associated with severe clinical manifestations. Majority of the infections were associated with CMV, which may raise susceptibility to other infections, CMV might not be an etiological agent, and its occurrence can be reactivation, prolonged shedding of carrier state or be due to coincidental infection. This observation on co-infection of respiratory viruses in children was supported by Zimmerman *et al.* and Esposito *et al.* [21] and is most likely due to their increased susceptibility to viral infection.

In this study, we identified and characterized viral pathogens to determine the etiology of acute respiratory infections including the zoonotic respiratory viruses which are poorly characterized. The positive detection rate of respiratory viruses was 49.0 %, with 8.5 % co-detection rate. The most common viruses detected in all ages were *Herpesviridae*, *Enterovirus*, influenza A virus and RSV. In addition to the influenza virus routinely screened in ARTI studies, other viral agents associated with severity like *Herpesviridae*, *Enterovirus* and RSV should be screened in respiratory illnesses. The study sheds lights on the viral spectrum of ARTI in children and adults in Tabanan Regency, Bali. Additional studies are required to determine nationwide epidemiology of respiratory viruses and association between viruses and clinical severity to guide prevention strategies in Indonesia.
